# A systematic review protocol for identifying the effectiveness of greenhouse gas mitigation interventions for health care systems in low- and middle-income countries

**DOI:** 10.12688/wellcomeopenres.18005.1

**Published:** 2022-08-04

**Authors:** Iris Martine Blom, Javier Shafick Asfura, Mohamed Eissa, Juliette Claudine Mattijsen, Hamaiyal Sana, Andrew Haines, Sarah Whitmee

**Affiliations:** 1 Population Health, London School of Hygiene & Tropical Medicine, London, UK; 2 National Autonomous University of Honduras, Tegucigalpa, Honduras; 3 Alexandria Faculty of Medicine, Alexandria, Egypt; 4 Erasmus University Rotterdam, Rotterdam, The Netherlands; 5 Bolan University of Medical & Health Sciences, Quetta, Pakistan; 6 Centre on Climate Change and Planetary, London School of Hygiene & Tropical Medicine, London, UK

**Keywords:** climate change, planetary health, health systems, sustainability, resilience, mitigation, adaptation

## Abstract

**Background**: Climate change is predicted to be our century's most significant health threat. In 2021, 46 countries committed to environmentally sustainable low carbon health care systems. Of those, 34 were from low- and middle-income countries (LMICs). Currently, health systems are responsible for 4.4% of global greenhouse gas (GHG) emissions, with health systems in high-income countries (HICs) contributing the largest proportion to the sector's GHG emissions. However, future increases are predicted in LMICs in the absence of robust GHG mitigation. This systematic review aims to identify evidence-based GHG mitigation interventions to guide the transformation of health care systems towards net zero, specifically in LMICs. Additionally, potential synergies between interventions that aid adaption to climate change and mitigate GHG emissions will be investigated.

**Methods**: This protocol will follow the 'Preferred Reporting Items for Systematic review and Meta-Analysis Protocols (PRISMA-P) checklist of recommended items to address in a systematic review protocol'. A comprehensive search will be conducted on electronic databases identified as relevant. Search terms were identified to capture all relevant peer-reviewed, primary research published between 1990 and 2022. The risk of bias will be assessed, and the quality of evidence graded. The eventual narrative synthesis will feed into a theory of change framework on GHG mitigation of health care systems in LMICs.

**Discussion**: This systematic review will synthesise the existing evidence around GHG mitigation interventions across all scopes of emissions, including scope 1 (health care operations), scope 2 (energy), and scope 3 (supply chains). It can be used to inform recommendations on how health care systems in LMICs can reduce emissions while prioritising which actions to take to gain the most significant reductions in GHG emissions, considering ease of implementation, scope and cost. Finally, this can catalyse further research in this area which is urgently needed.

## Introduction

Without action to reduce global greenhouse gas (GHG) emissions, climate change is predicted to be the biggest threat to global public health in the 21st century due to many direct and indirect health effects, including extreme weather, the spread of vector-borne diseases, lack of access to clean water and mental health impacts ^1^. Although health care systems will have to deal with the health impacts of this looming public health crisis, they are also responsible for 4.4% of GHG emissions globally, thereby contributing to it ^2^. At the United Nations Framework Convention on Climate Change 26 ^th^ Conference of Parties (UNFCCC COP26) in November 2021, 46 countries committed to a transition to sustainable, low carbon health systems defined by the WHO as systems that improve, maintain or restore health while minimising negative impacts on the environment and leveraging opportunities to restore and improve it, for the benefit of the health and well-being of current and future generations ^3, 4^. Furthermore, 14 countries committed to achieving net-zero health systems between 2030 and 2050 ^4^. Among the countries pledging, many were low- and middle-income countries (LMICs), namely 34 and 11, respectively ^4^. Even though health care systems in LMICs have lower GHG emissions than high-income countries (HICs), as health care systems in many LMICs advance, an increase in these emissions is expected unless action is taken to identify, quantify and reduce them. In addition, LMICs are expected to experience the negative impacts on health from climate change both earlier and most severely due to geographical location and exposure, whilst being the least equipped to deal with them because of lack of resources to cope and recover ^5^. It is vital to ensure that any adaptation actions undertaken by health care systems do not also exacerbate the sector's GHG emissions, creating feedback loops locking them into higher-emission trajectories. However, there is a current gap in knowledge on transforming health care systems in LMICs to adapt to climate change while transitioning to low carbon. Therefore, to bring the COP26 commitments to reality, evidence-based GHG mitigation interventions towards more sustainable health care systems in LMICs must be identified across all scopes of emissions including scope 1 (health care operations), scope 2 (energy), and scope 3 (supply chains). This article will describe a systematic review protocol towards this aim following the Preferred Reporting Items for Systematic review and Meta-Analysis Protocols (PRISMA-P) checklist of recommended items toa address in a systematic review protocol ^6^.

### Aims, objectives and research questions

This systematic review aims to identify practical and theoretical GHG mitigation interventions for health care in LMICs. The following research questions guide this study and summarise its objectives:

1) What practical or theoretical GHG mitigation interventions across health care operations, energy, and supply chains can be identified that decrease greenhouse gas emissions in the context of low- and middle-income countries?
2) What are the implementation processes to reach the desired outcomes, including goal setting, determining roles and responsibilities, delegating tasks, execution and monitoring of tasks, and the evaluation; and what are enablers of and barriers to implementation?
3) How do the GHG mitigation interventions interact with actions to promote adaptation and resilience, including possible synergies, co-benefits, conflicts or trade-offs?
4) How do these interventions vary contextually, and what aspects are applicable across different contexts? Contextual variables include the economic context ( *e.g.* economic growth, unemployment rate), the socio-cultural context ( *e.g.* social values, religion), and the political-legal context ( political stability, legal framework).

## Methods

A systematic review will be undertaken to collate, critically appraise and synthesise existing evidence on practical or theoretical GHG mitigation interventions across health care operations, energy and supply chains in the context of LMICs. Various aspects will be explored, including the implementation process. Furthermore, the relation of these interventions with adaptation will be analysed where reported. Within the following paragraphs, different aspects of the methodology will be discussed.

### Eligibility criteria

Table 1 shows the areas considered in screening the articles and the related inclusion and exclusion criteria.

**Table 1.** Inclusion and exclusion criteria of the systematic review.

Area	Criteria
Publication type	Only peer-reviewed primary research will be included, including analytical cross-sectional studies, case-control studies, case reports, cohort studies, diagnostic test accuracy studies, and randomised controlled trials. Any other articles, such as protocols, guidelines, (systematic) reviews, perspectives, commentaries, or editorials, will be excluded. However, relevant reviews will be screened for primary references.
Language	Articles written in English, Spanish, Italian, Portuguese, French and Arabic will be included for screening. All other languages will be excluded.
Context	Only articles will be included from which the context of the research is in LMICs. It will be excluded if the research context is in HICs, or general and not specific to a country, group of countries or region.
Topic	Only articles will be included that mention any theoretical or practical GHG mitigation intervention across health care operations, energy and supply chains towards a decrease of GHG emissions. Articles that do not report such a mitigation intervention will be excluded.
Metrics	Only articles that report a quantified change in GHG emissions from the intervention as mentioned above will be included. If a measurable outcome is not reported, the article will be excluded.
Timeline	Only articles published between 1990 and 30 January 2022, will be included. 1990 is chosen as a starting point for the inclusion of articles since it is the start of a significant research movement supporting the climate change and health connection ^8^. Articles that were written before 1990 are excluded.

### Information sources

This systematic review will make use of electronic databases as information sources. The electronic databases that have been evaluated to be relevant and intended to be searched for the systematic review are Ovid MEDLINE, Ovid EMBASE, Global Health, SCOPUS, Web of Science, AfricaPortal, Africa-Wide Information, LILACS, Global Index Medicus, GreenFILE and ELDIS.

### Search strategy

A broad and sensitive search strategy has been designed, which will be repeated across the identified relevant databases. Table 2 includes a specific example of the search strategy that has been drafted for the electronic database Ovid MEDLINE.

**Table 2.** Search strategy of the systematic review drafted for the electronic database Ovid MEDLINE.

Search line	Content of search
1	(netzero or net zero).mp.
2	Carbon Footprint/
3	Greenhouse Effect/
4	exp Climate Change/
5	(carbon or CO2 or methane or CH4 or nitrous oxide or nitrus oxide or N2O or hydrofluorocarbon* or HFC* or perfluorocarbon* or PFC* or F-gas or fluorinated gas or sulfur hexafluoride or SF6 or nitrogen trifluoride or NF3 or emission* or greenhouse or GHG or climat* change* or global warming or footprint or eco-friendly or climate friendly or environment* friendly or eco-efficient or environment* responsible or environment* sound or energy-efficient or energy-saving or green initiative* or environmental impact or short-lived climate pollutant or black carbon).mp.
6	(environment* and sustainab*).mp.
7	1 or 2 or 3 or 4 or 5 or 6
8	exp "Delivery of Health Care"/
9	exp Health Facilities/
10	(health system* or health-care or health-care or health sector or health supply chain* or health service* or delivery of health or health delivery or health facilit* or health cent* or hospital or hospitals or clinic or clinics or emergency room* or operat* room* or operat* theat* or patient care or ward* or urgent care or primary care or secondary care or tertiary care or quaternary care or telemedicine or medical cent* or diagnostic care or rehabilitative care or preventative care or palliative care or home care).mp.
11	8 or 9 or 10
12	7 and 11
304	or/13-303 [ALL LOW AND MIDDLE-INCOME COUNTRIES (expert search)]
305	12 and 304
306	limit 305 to yr="1990 - 2022"

### Study records

***Data management.*** The references of the articles identified through the search strategies on the relevant electronic databases will be uploaded to the software Rayyan QCRI which allows simultaneous collaboration between all screeners. The inclusion and exclusion criteria will be applied in every step of the screening process as outlined below. Citation, abstracts and full articles will be uploaded to be used at the different, relevant screening steps. Every screener unfamiliar with the software will receive a training session from the first author to gain familiarity with its use.

***Selection process.*** After removing duplicates, papers will be initially screened by title, following Mateen *et al.*'s recommendations to improve the screening process's efficiency ^7^. Then, articles will be screened by abstract and shortlisted articles will be screened through full-text analysis against eligibility criteria using the software Rayyan QCRI. At least two reviewers will perform each screening step, and any disagreements regarding inclusion will be discussed. If there is no consensus between two screeners, a third author will be consulted until an agreement is reached.

***Data collection process.*** Data from eligible articles will be collated independently using a tailored data collection form with a detailed instruction manual trialled before use. As part of the pilot phase, four reviewers will extract data from the same five articles, after which the form will be discussed and adjusted based on experience and feedback. This will also contribute to improved consistency of data collection between different reviewers.

***Data items.*** Table 3 shows an overview of the data items for which data will be sought.

**Table 3.** List of variables for which data will be sought as part of the systematic review. GHG: Greenhouse gas.

Data item	Definition
Article identifiers	Basic identifiers of the article will be extracted, including name, authors, date, journal, article type and article design.
Methodology	The methodology used in the article will be identified and extracted.
Geographical scale	The geographical scale, namely if it was conducted at the local, regional, national or international level.
Location	The article's location will be extracted by identifying the relevant town/city, region, country and/or countries where the research was conducted.
Emission scope	If a particular emission scope was researched, this will be extracted, and it will be identified whether the research interacts with scope 1 (health care operations), scope 2 (energy), scope 3 (supply chains) or multiple scopes.
Part of the health care system	If a particular aspect of the health care system was researched, this will be extracted, ( *e.g.* a primary health care clinic, a rural hospital).
GHG mitigation intervention(s)	The GHG mitigation intervention(s) are the interventions that lead towards a decrease in GHG emissions, including its details.
Measurable impact of the GHG mitigation intervention(s)	The quantified impact of the identified intervention(s) of the research on mitigation, including a specification of GHG or GHG equivalent and whether it is a practical or theoretical impact.
Implementation process	The implementation process will be extracted, including enablers and barriers that were faced and how these were or will be approached.
Implementation timeline	The timeline around the implementation will be extracted in terms of length around the implementation process.
Economic analysis	If included, the economic aspects such as cost effectiveness, cost benefit or cost consequences will be extracted.
Linkage with adaptation or resilience	If the intervention is directed at both mitigation and adaptation or specifically resilience is described, this will be extracted. These interactions can be synergies, co-benefits, conflicts, trade-offs or co-harms ^9^.
Health impact	If the intervention has a measured impact on health outcomes or exposures, this will be extracted.
Funding source	The source of funding for the authors will be extracted to identify potential conflicts of interest.
Conflicts of interest	Further potential conflicts of interest will be extracted, including relationships with relevant parties other than financial relationships.
Summary	Each article will be summarised in under 100 words on the extraction sheet.

### Outcomes and prioritisation

The primary outcome is the identification of GHG mitigation interventions undertaken with the aim of reducing GHG emissions within health care systems in the context of LMICs and the quantified emission reductions associated with each mitigation action. The main objective of the research is to identify these interventions as there is a lack of overview of evidence-based interventions towards environmental sustainability in this context.

Secondary outcomes include identifying links with climate change adaptation actions, including climate resilience, the emission scope of the intervention, and the implementation process, including the timeline and enablers or barriers faced. The collection of other secondary outcomes is pertinent to inform policy recommendations regarding which interventions will be easiest to implement and in which context, and where actions can be scaled or translated between different contexts.

### Risk of bias in individual studies

For each included article, the risk of bias and quality will be assessed using the Joanna Briggs Institute (JBI) Critical Appraisal Tools. These tools are designed across multiple study types and are therefore applicable to this systematic review. While using the tool for assessment, the assessor will describe verbatim quotes. Based on the extracted information, the risk of bias will be assessed as low, medium or high. An assessment will be 'unclear' if relevant information is missing from the assessed article. The assessments will be made independently by at least two authors, after which they will be compared. Any disagreements will be discussed, and a third author will be consulted if no consensus is found. The risk of bias in each included article will be reported in the eventual manuscript of the systematic review ^10^.

### Data synthesis

It is unlikely that extracted data from included articles in this systematic review will be appropriate for quantitative synthesis because of the diversity of contexts, types and scale of intervention and possible outcomes. A narrative synthesis will present the identified data of the included articles. A table will be provided to summarise the included articles and their findings to facilitate this synthesis. Findings will be grouped by type of intervention where possible. Through narrative analysis, these findings will be further explored and compared between articles. Furthermore, the identified data will feed into a theory of change theoretical framework on GHG mitigation interventions for health care systems in LMICs.

### Meta-bias(es)

Reporting bias will be investigated by recording whether included articles are proceeded by a protocol published before the article's publication. If selective reporting of the results is identified while comparing the protocol to the eventual article, this will be reported.

### Confidence in cumulative evidence

To assess the overall strength of the body of evidence created from the synthesis of the included articles, the evidence will be graded using the approach developed by the Grading of Recommendations Assessment, Development and Evaluation (GRADE) Working Group. This tool includes the domains 'Risk of Bias', 'Imprecision', 'Inconsistency', 'Indirectness', and 'Publication Bias'. The eventual evidence will be graded using four different categories. As described by Siemieniuk *et al.*, these categories are that the certainty of the evidence is 1) very low (the true effect is probably very different from the estimated effect), 2) low (the true effect might be very different from the estimated effect), 3) moderate (the authors believe that the true effect is probably close to the estimated effect) or 4) high (the authors are confident that the true effect is similar to the estimated effect) ^11^.

### Dissemination of information

The findings and outcomes of this study will be published in peer-reviewed scientific journals and presented at conferences and meetings related to planetary health, climate change and health, and health systems. The findings will also be disseminated to the broader public using a social media dissemination strategy.

### Amendments

This protocol is the first publication. In case of important protocol amendments following review, they will be tracked, dated and published as such on Wellcome Open Research.

## Discussion

Climate change is expected to have a major impact on health ^1^. While health care systems need to become prepared to deal with these health effects, they must also move to sustainable practice to halt their contribution to this health emergency. Most countries committed to sustainable health care systems at COP26 are LMICs, yet there is a lack of structured evidence to inform policy ^4^. Furthermore, health system research rarely considers the interaction between these GHG mitigation interventions and adaptation, which is especially important in vulnerable locations. To respond to this emergency, this protocol describes the approach to a systematic review which will provide an overview of the current existing peer-reviewed evidence on interventions towards GHG mitigation of health care systems in LMICs. To the authors' knowledge, this will be the first attempt to create this overview. Given the urgency around climate change and its impact on health, it is also a timely one. It will provide the first step in the direction of evidence-based guidance toward GHG mitigation of health care systems in LMICs.

Several potential sources for biases for this review, common to this methodology, could impact the quality of the evidence presented in the eventual synthesis. First, the risk of publication bias must be considered for three reasons. The first reason is that GHG mitigation research is a recent area of research that is rapidly developing and expanding, considering the topic's urgency. It could be regarded as likely that not all successful mitigation interventions are indeed published in peer-reviewed journals due to the perceived lengthy publication process. The second reason is that interventions with a measured impact are more likely to be published than those with lesser or no significant impact on decreasing GHG emissions. The final reason that might contribute to publication bias is that certain areas of mitigation, such as those that produce scope 1 and 2 emissions, might receive more research funding than emissions from scope 3. The publication bias will be assessed as part of the synthesis during the systematic review.

A second potential bias to consider is the reviewer bias which can be caused by varied interpretations of inclusion criteria by different reviewers. To reduce this risk of bias, all reviewers will be trained and familiarized with the program before starting. Furthermore, each article will be screened by at least two reviewers during every step of article screening. Any disputes will be discussed, and a third reviewer will be involved if no consensus can be reached.

Finally, a third potential bias to be aware of during the process of this systematic review is the existence of inconsistent terms and definitions. In the relatively young area of research into GHG mitigation, terms are used interchangeably and often not clearly defined. To reduce this risk of bias, the search strategy is broad and includes a wide range of terms that can be relevant to the research topic.

As outlined above, the search strategy aims to be comprehensive. Therefore, a challenge during the synthesis might be that heterogeneity of reporting styles is observed between the selected articles: for example, using various metrics and units across contexts. The authors will aim to translate heterogeneous results to allow for quantitative synthesis and interpretation, where possible.

In conclusion, this protocol describes a systematic review methodology that aims to provide an urgently needed overview of interventions toward GHG mitigation in health care systems. Furthermore, any connections with climate change adaptation by health care systems will also be synthesised. Through that, the review will have the opportunity to contribute to ongoing GHG mitigation and adaptation efforts. Furthermore, doing so will also contribute to identifying areas where more research is needed to guide future efforts in an evidence-based manner. 

## Data availability

### Underlying data

No data are associated with this article.

### Reporting guidelines

Medicine: PRISMA-P Checklist for ‘A Systematic Review Protocol for Identifying the Effectiveness of Greenhouse Gas Mitigation Interventions for Health Care Systems in Low- and Middle-Income Countries’, https://doi.org/10.17037/DATA.00002988

Data are available under the terms of the Creative Commons Zero "No rights reserved" data waiver (CC0 1.0 Public domain dedication).
