# Supplementary material for: A systematic review protocol for identifying the effectiveness of greenhouse gas mitigation interventions for health care systems in low- and middle-income countries
Source: Wellcome Open Res. 2023 Jun 12;7:202. Originally published 2022 Aug 4. [Version 2] doi: 10.12688/wellcomeopenres.18005.2 (PMC11004597; doi:10.12688/wellcomeopenres.18005.2)
Supplement: Peer review history

# Reviewer report: Reviewer response for version 2

**DOI:** 10.21956/wellcomeopenres.21670.r77116

**Published:** 2024-06-06

**Authors:** Cristina Richie

**Affiliations:** 1 The University of Edinburgh, Edinburgh, Scotland, UK

The protocol seems sufficient and should consider ethics as well. I look forward to seeing it implemented.

 Ethical factors include remediating the social determinants of health; the obligation for distributive health care justice; and the environmental imperative to minimize carbon emissions. All these point to the need to reallocate health care carbon away from luxury and high tech goods to basic health care for all.

Is the study design appropriate for the research question?

Yes

Is the rationale for, and objectives of, the study clearly described?

Yes

Are sufficient details of the methods provided to allow replication by others?

Yes

Are the datasets clearly presented in a useable and accessible format?

Yes

Reviewer Expertise:

ethics; sustainable health care; decarbonization of health care; biomedical ethics

I confirm that I have read this submission and believe that I have an appropriate level of expertise to confirm that it is of an acceptable scientific standard.

## References

[1] : Principles of green bioethics: sustainability in health care. *Michigan State University Press* .2019; Reference source

[2] : Priority setting and net zero healthcare: how much health can a tonne of carbon buy?. *BMJ* .2021;375: 10.1136/bmj-2021-067199 e067199 10.1136/bmj-2021-067199 34711584

# Reviewer report: Reviewer response for version 2

**DOI:** 10.21956/wellcomeopenres.21670.r77112

**Published:** 2024-04-11

**Authors:** Zerina Lokmic Tomkins

**Affiliations:** 1 Monash University, Melbourne, Victoria, Australia

Thank you for the opportunity to review this work. There are only few comments for authors to consider:

 Abstract:

 It is not clear in the abstract if the review is using qualitative, quantitative or mixed methods approach -please consider clarifying this.

 Methodology:

 How will the effectiveness of interventions be evaluated - is it only going to be described or will it also be assessed on what has worked/not worked - consider if using template for intervention description and replication (TiDiER) checklist might be more appropriate to report the findings as a complement to your own tool. Although mostly used to report clinical interventions, the tool is adaptable to other settings. Using TiDiER might help reduce some of the biases described in this project proposal. 

 I was also curious to understand how will you clarify between mitigation and adaptation/resilience variables ( Table 3) - are specific indicators used to distinguish between the two.

Is the study design appropriate for the research question?

Yes

Is the rationale for, and objectives of, the study clearly described?

Yes

Are sufficient details of the methods provided to allow replication by others?

Partly

Are the datasets clearly presented in a useable and accessible format?

Yes

Reviewer Expertise:

Environmental health, climate change adaptation (maternal and child health) digital health

I confirm that I have read this submission and believe that I have an appropriate level of expertise to confirm that it is of an acceptable scientific standard, however I have significant reservations, as outlined above.

## References

[1] : Better reporting of interventions: template for intervention description and replication (TIDieR) checklist and guide. *BMJ* .2014;348: 10.1136/bmj.g1687 g1687 10.1136/bmj.g1687 24609605

# Reviewer report: Reviewer response for version 2

**DOI:** 10.21956/wellcomeopenres.21670.r77110

**Published:** 2024-04-11

**Authors:** Caleb Dresser

**Affiliations:** 1 Beth Israel Deaconess Medical Center / Harvard Medical School, Boston, USA

This systematic review protocol appears well-designed and the team appears to be prepared to execute on this plan effectively. 

 The authors plan to conduct a systematic review of the published literature on GHG emissions reduction in low and middle income country healthcare systems. Their inclusion of both medically focused databases (their first five) and LMIC-focused databases (their second five) is laudable; while this will present a substantial screening challenge given the volume of articles that may be produced on initial searches, this will also provide the most comprehensive possible list of articles for consideration for inclusion.

 I agree with the author's assessment of the current state of the literature in this field, and agree that a narrative synthesis is likely to be the most effective (and most feasible) approach for interpreting the literature that is identified. Uneven use of terminology in this evolving field will present a challenge, and it is highly unlikely that the articles identified in this search will be sufficiently congruent in terms of methods or reported outcomes to be suitable for formal meta-analysis.

 The protocol appears well-designed, and will contribute useful new information to this field.

Is the study design appropriate for the research question?

Yes

Is the rationale for, and objectives of, the study clearly described?

Yes

Are sufficient details of the methods provided to allow replication by others?

Yes

Are the datasets clearly presented in a useable and accessible format?

Yes

Reviewer Expertise:

My work focuses on climate change and health, with a focus on health systems and the implications of climate change for healthcare. While the majority of my work focuses on healthcare-based adaptation to climate change, I also serve on our hospital's sustainability committee, teach a course at Harvard's school of public health that covers healthcare sustainability in the context of climate change, and have designed and conducted multiple systematic reviews in the field of climate change and health.

I confirm that I have read this submission and believe that I have an appropriate level of expertise to confirm that it is of an acceptable scientific standard.

# Reviewer report: Reviewer response for version 2

**DOI:** 10.21956/wellcomeopenres.21670.r77107

**Published:** 2024-04-09

**Authors:** Claudia Quitmann

**Affiliations:** 1 University Hospital Heidelberg and Medical Faculty of Heidelberg University, Heidelberg Institute of Global Health, Heidelberg, Germany

Thank you for giving me the opportunity to review the revised version of: "A systematic review protocol for identifying the effectiveness of greenhouse gas mitigation interventions for health care systems in low- and middle-income countries".

 Some minor comments:

**Abstract:**

 - In 2021, 46 countries committed to environmentally sustainable low carbon health care systems. Please refer to a more recent year.

 - Mention number of databases in the abstract

 - scope 1 (health care operations) health care operation sounds a bit misleading and scope 1 emissions can also be energy-related; I suggest rephrasing the words in the brackets to more closely follow the GHG Protocol. 

 - Search terms were identified to capture all relevant peer-reviewed, primary research published between 1990 and 2022. The amendment states that articles are included until March 2023 - please correct abstract.

**Introduction:**

 - 1) What practical or theoretical GHG mitigation interventions across health care operations, energy, and supply chains can be identified that decrease greenhouse gas emissions in the context of low- and middle-income countries? I suggest to use the terms scope 1-3.

 - Is it planned to analyze objective 2-4 by intervention? I assume that the results differ quite a lot depending on the intervention (e.g., building insulation vs. plant-based menus vs. abolishing desflurane).

**Methods:**

 - Only peer-reviewed primary research will be included. I assume that grey literature would be a very valuable source, please state briefly why it was decided to exclude it.

 I wish the authors all the best for their scoping review!

Is the study design appropriate for the research question?

Yes

Is the rationale for, and objectives of, the study clearly described?

Yes

Are sufficient details of the methods provided to allow replication by others?

Yes

Are the datasets clearly presented in a useable and accessible format?

Not applicable

Reviewer Expertise:

Planetary Health

I confirm that I have read this submission and believe that I have an appropriate level of expertise to confirm that it is of an acceptable scientific standard, however I have significant reservations, as outlined above.

# Reviewer report: Reviewer response for version 2

**DOI:** 10.21956/wellcomeopenres.21670.r59939

**Published:** 2023-06-19

**Authors:** Raanan Raz

**Affiliations:** 1 Braun School of Public Health and Community Medicine, The Hebrew University of Jerusalem, Jerusalem, Israel

I approve this article for indexing

Is the study design appropriate for the research question?

Yes

Is the rationale for, and objectives of, the study clearly described?

Yes

Are sufficient details of the methods provided to allow replication by others?

Yes

Are the datasets clearly presented in a useable and accessible format?

Not applicable

Reviewer Expertise:

NA

I confirm that I have read this submission and believe that I have an appropriate level of expertise to confirm that it is of an acceptable scientific standard.

# Reviewer report: Reviewer response for version 1

**DOI:** 10.21956/wellcomeopenres.19964.r55482

**Published:** 2023-04-11

**Authors:** Raanan Raz

**Affiliations:** 1 Braun School of Public Health and Community Medicine, The Hebrew University of Jerusalem, Jerusalem, Israel

This vital study is aimed to systematically evaluate methods of GHG emission reduction in the health sector in LMIC countries. With this, it reflects a real need to examine which of the efforts in this area is thriving and efficient, the various enablers and barriers for implementation, the scope and magnitude of the research in this field, and many other issues that have yet to be systematically addressed. The researchers strictly adopt a systematic review approach using the PRISMA-P checklist, avoiding and assessing biases as much as possible. This is an excellent protocol for this task, and this timely research can certainly help direct precious efforts toward the most efficient approaches.

 With this, I have one major comment for the researchers to consider. The described protocol considers only peer-reviewed articles, as often when systematically reviewing a scientific health issue. However, many implementations of GHG emission reduction in the health sector, particularly in LMIC countries, are not necessarily accompanied by peer-reviewed articles. This is because some of these projects may lack substantial research aspects and implement relatively simple and obvious interventions. Such projects are not documented in peer-reviewed articles; instead, they may be published in various ways by national and international organizations, such as GGHH ( https://greenhospitals.org/) and others. Therefore, I encourage the research team to broaden their search to include other sources of information about relevant projects in LMIC.

Is the study design appropriate for the research question?

Yes

Is the rationale for, and objectives of, the study clearly described?

Yes

Are sufficient details of the methods provided to allow replication by others?

Yes

Are the datasets clearly presented in a useable and accessible format?

Not applicable

Reviewer Expertise:

Environmental Epidemiology

I confirm that I have read this submission and believe that I have an appropriate level of expertise to confirm that it is of an acceptable scientific standard.

# Response

**Published:** 2023-06-07

**Authors:** Iris Blom

**Affiliations:** London School of Hygiene & Tropical Medicine, UK

Dear Reviewer,   Thank you for taking the time to review our manuscript and for your valuable comments. We are delighted to learn that you perceive our study's relevance and potential impact.  Regarding your major comment about considering non-peer-reviewed articles and grey literature, we wholeheartedly agree with your insights. Many successful GHG emission reduction projects in the health sector, especially in LMIC countries, may not be documented in peer-reviewed articles, but rather, they might be published through national and international organizations. Your suggestion to broaden our search criteria to include these additional sources of information is both valid and valuable. While the current scope of our review is to provide a comprehensive overview of what is known within peer-reviewed literature, we understand that this may present a limitation in the study. To address this, we will ensure to clearly outline this limitation in our manuscript and emphasize the need for including an understanding of the grey literature as a significant recommendation for future research. This will be noted as a crucial next step in further research, with the hope to eventually capture a broader and more realistic range of GHG emission reduction strategies in the health sector across LMICs. We are grateful for your constructive feedback, which will greatly enhance our work.   With kind regards, Dr Iris Martine Blom

# Reviewer report: Reviewer response for version 1

**DOI:** 10.21956/wellcomeopenres.19964.r54136

**Published:** 2023-03-10

**Authors:** Francis D. Pope

**Affiliations:** 1 School of Geography, Earth and Environmental Sciences, University of Birmingham, Birmingham, UK

The protocol details a systematic review for identifying the effectiveness of GHG mitigation interventions for health care systems within LMICs. The subject area is timely, with the health care sector responsible for almost 5% of global GHG emissions.

 Overall, the protocol is sensible looking at relevant articles between 1990 and 2022. The review will be a narrative review because of the lack of similar studies with which to get quantitative comparison out of. The protocol should be indexed once the following comments have been responded to.

 Within the introduction, feedback loops between GHG emissions and the health care sector are discussed. It is not clear how these feedback loops will be set up.

 The review will look at scope 1, scope 2 and scope 3 emissions. Scope 3 is respect to supply chains. There is a huge literature with respect to vehicle emissions most of which will not mention the health care sector.  How will you ensure you capture the relevant information from the wide range of literatures that will need to be read?

 Similarly, with the respect to “…promote adaptation and resilience, including possible synergies, co-benefits, conflicts and trade offs.”  Much of this literature will come from outside the healthcare literature.  A plan to capture this information will be required.

 The information sources discussed in the search strategy should be justified. Detail what each database will bring and how it is distinct from the others.

 The language of the papers that will be reviewed is wide, and this is to be commended. Does the team have the required language skills to review all these languages?  If not, how will this be dealt with.

 Timeline – all articles within 2022 should now be possible to review, not just those up to 30 Jan 2022.

Is the study design appropriate for the research question?

Yes

Is the rationale for, and objectives of, the study clearly described?

Yes

Are sufficient details of the methods provided to allow replication by others?

Yes

Are the datasets clearly presented in a useable and accessible format?

Not applicable

Reviewer Expertise:

Climate change and green house gas emissions.

I confirm that I have read this submission and believe that I have an appropriate level of expertise to confirm that it is of an acceptable scientific standard, however I have significant reservations, as outlined above.

# Response

**Published:** 2023-06-07

**Authors:** Iris Blom

**Affiliations:** London School of Hygiene & Tropical Medicine, UK

Dear Reviewer,   Thank you for the time and effort you have dedicated to reviewing our manuscript. Your comments and insights are greatly appreciated, and we believe they significantly contribute to the improvement and clarification of our work. We have carefully considered your feedback and addressed each of your comments as detailed below: Regarding the lack of clarity on feedback loops between GHG emissions and the healthcare sector, we appreciate your concern. In light of your comment, we have revised the wording in this section to better express our intended meaning. Specifically, we mean to convey that if adaptation measures that increase greenhouse gases are implemented, they could negatively impact mitigation efforts. Your point about the challenges associated with capturing relevant information from the vast literature on vehicle emissions, especially those not explicitly referencing the healthcare sector, is indeed crucial. We agree that the healthcare system transformation requires learning extensively from efforts across all sectors due to its interconnected nature, especially concerning scope 3 emissions. Our primary goal for this systematic review is to provide a comprehensive overview of research conducted specifically within the healthcare context. We understand the potential limitations of this focus and will ensure they are clearly stated in our study. As for the concern about capturing literature from outside the healthcare sector related to promoting adaptation and resilience, our approach will be akin to the one mentioned above. While we strive to create a thorough understanding of what is known within the healthcare systems context, we will be explicit about the limitations of this approach in our study. We appreciate your suggestion about justifying the information sources discussed in our search strategy. We have now included explanations for the selection the databases in the methodology, outlining their unique contributions to our study. Thank you for acknowledging the range of languages in which we aim to review papers. We can confirm that our team has the necessary skills to review papers in all the languages listed. Lastly, in response to your note on the timeline, we have revised the manuscript to include all articles up till March this year, thus extending beyond the initially proposed date of 30th January 2022. We hope that our revisions and responses adequately address your comments and concerns. We look forward to hearing from you regarding the next steps in the review process. Thank you again for your careful reading and valuable feedback.   With kind regards, Dr Iris Martine Blom
